# Immobilization and Characterization of a Processive Endoglucanase EG5C-1 from *Bacillus subtilis* on Melamine–Glutaraldehyde Dendrimer-Functionalized Magnetic Nanoparticles

**DOI:** 10.3390/nano14040340

**Published:** 2024-02-09

**Authors:** Xiaozhou Li, Jie Chen, Bin Wu, Zhen Gao, Bingfang He

**Affiliations:** 1 College of Biotechnology and Pharmaceutical Engineering, Nanjing Tech University, Nanjing 211800, China; 202161218101@njtech.edu.cn (X.L.); 202261118054@njtech.edu.cn (J.C.); wubin1977@njtech.edu.cn (B.W.); 2 School of Pharmaceutical Sciences, Nanjing Tech University, Nanjing 211800, China; bingfanghe@njtech.edu.cn

**Keywords:** processive endoglucanase, immobilization, magnetic nanoparticles, melamine–glutaraldehyde dendrimer

## Abstract

Exploring an appropriate immobilization approach to enhance catalytic activity and reusability of cellulase is of great importance to reduce the price of enzymes and promote the industrialization of cellulose-derived biochemicals. In this study, Fe_3_O_4_ magnetic nanoparticles (MNPs) were functionalized with meso-2,3-dimercaptosuccinic acid to introduce carboxyl groups on the surface (DMNPs). Then, melamine–glutaraldehyde dendrimer-like polymers were grafted on DMNPs to increase protein binding sites for the immobilization of processive endoglucanase EG5C-1. Moreover, this dendrimer-like structure was beneficial to protect the conformation of EG5C-1 and facilitate the interaction between substrate and active center. The loading capacity of the functionalized copolymers (MG-DMNPs) for EG5C-1 was about 195 mg/g, where more than 90% of the activity was recovered. Immobilized EG5C-1 exhibited improved thermal stability and increased tolerability over a broad pH range compared with the free one. Additionally, MG-DMNP/EG5C-1 biocomposite maintained approximately 80% of its initial hydrolysis productivity after five cycles of usage using filter paper as the substrate. Our results provided a promising approach for the functionalization of MNPs, enabling the immobilization of cellulases with a high loading capacity and excellent activity recovery.

## 1. Introduction

The conversion of cellulose into fermentable sugars through cellulase hydrolysis constitutes a pivotal role for the production of biofuels and platform chemicals from cellulosic biomass [1,2,3]. However, the exorbitant price, high loading required in this enzymatic reaction, and limited stability against the harsh environment of cellulases greatly impede the industrial-scale application of the biomass conversion. Immobilization of enzymes onto solid supports provides numerous advantages, including the enzyme reusability, easy separation of product, and enhanced enzyme stability, which substantially reduce the cost of an enzyme in commercial applications [4,5]. Therefore, various technologies to date have been developed for cellulase immobilization, including adsorption, entrapment, cross-linking, and covalent binding [6,7]. Among these approaches, covalent binding is considered a highly effective way for cellulase immobilization due to strong interactions between enzyme and support, which endows the cellulase more stable and reusable [8,9].

Since cellulose is not soluble in water, the hydrolysis of cellulose catalyzed by cellulases is a typical heterogeneous reaction. When cellulases are immobilized on insoluble supports, the hydrolysis efficiency of cellulose is often low in view of the limited accessibility of the enzyme to the insoluble substrate. Therefore, the physicochemical properties of solid support play a vital role on the catalytic performance of immobilized cellulase [10]. Hitherto, a variety of solid supports, such as inorganic materials (mesoporous silica, zeolite, and graphene oxide), organic polymers (chitosan microsphere, methacrylic acid, methyl methacrylate copolymer, and styrene/maleic anhydride copolymer), nanomaterials (nanoparticle, nanotube, and nanofiber), and metal-organic frameworks (MOF), have been developed to immobilize cellulases [11,12,13,14]. Recently, magnetic nanoparticles have received extensive attention in cellulase immobilization, owing to their remarkable properties, such as large specific surface area and an excellent dispersion property [15]. Since cellulose is insoluble, the outstanding dispersion property of nanoparticles in reaction medium can ensure a higher proportion of immobilized cellulase bonds to insoluble cellulose compared to bulk supports, which is particularly advantageous for cellulase immobilization [16]. Furthermore, nanoparticles with magnetic characteristics are easily recovered from the reaction mixture containing unhydrolyzed solid residues by applying an external magnetic field [17]. As a representative magnetic nanomaterial, Fe_3_O_4_ nanoparticles possess the aforementioned desirable characteristics as well as good biocompatibility, low cost, and simple preparation. However, surface modification is crucial for Fe_3_O_4_ nanoparticles to efficiently immobilize cellulase due to the absence of related functional groups [18]. Therefore, functionalization with amino, silica, chitosan, and surfactants have been used for cellulase immobilization onto iron oxide nanoparticles. For instance, Alftren et al. studied the immobilization of cellulase CellicCTec2 onto amino-functionalized magnetic Fe_3_O_4_ nanoparticles via covalent binding [19]. Results showed that the maximum enzyme-binding capacity of the particles was 14.6 mg/g. Kumar et al. reported that iron oxide nanoparticles were modified with 3-aminopropyltriethoxysilane, and then the resultant particles were applied in the immobilization of cellulase from *Aspergillus niger* [20]. They found that the loading capacity was 71.5 mg/g. In another study, cellulase was covalently immobilized on chitosan-coated magnetic Fe_3_O_4_ nanoparticles using glutaraldehyde as the crosslinking reagent. The obtained date showed that approximately 106 mg of protein was cross-linked per gram of nanoparticles [21]. Nevertheless, iron nanoparticles are prone to aggregation in aqueous solution, leading to a reduced number of binding sites on the surface; therefore, the relatively low loading capacity limits the subsequent application of these methods in cellulase immobilization.

In order to further improve the enzyme content on the surface of the support, dendritic polymers have been recently developed to modify nanomaterials for enzyme immobilization. Consequently, dendrimer-coated nanomaterials can effectively protect the conformation of enzyme and provide more active groups on the surface, resulting in an enhanced protein-loading capacity and larger contact area between the substrates and enzyme. Modified microporous polystyrene with polyamidoamine was used for porcine pancreas lipase immobilization and the loading capacity was increased by 10% [22]. Han [23] performed a study for cellulase immobilization using the surface of magnetic Fe_3_O_4_ nanoparticles modified by dendritic polymer 4-arm-PEG-NH_2_. The loading of cellulase immobilized on GO@ Fe_3_O_4_@5k 4-arm-PEG-NH_2_ and GO@ Fe_3_O_4_@10K 4-arm-PEG-NH_2_ reached 429 mg/g and 575 mg/g, respectively. Despite the fact that these previous works have increased loading capacity and recyclability of enzyme, the preparation of such supports is often time-consuming, requires tedious procedures to functionalize the carrier material, and has high production costs. Therefore, there is still great demand to exploit novel dendrimer-like polymers with highly reactive sites for cellulase immobilization. Cellulase, a multi-enzyme system composed of cellobiohydrolase, endo-glucanase, and β-glucosidase, acts synergistically in the decomposition of cellulosic biomass [24]. Recently, processive endo-glucanases that possess both endo-/exo-bifunctionality have gained considerable interest. Due to their dual function, processive endo-glucanases are thought to be functionally equivalent to endo-glucanase and cellobiohydrolase that together comprise a traditional cellulase system. However, efforts to immobilize such an enzyme for improved stability and reusability are still scarce. In our previous work, a novel processive endo-glucanase EG5C-1 from *Bacillus subtilis* was identified and characterized [25].

In this study, Fe_3_O_4_ magnetic nanoparticles were initially synthesized by a co-precipitation method and subsequently subjected to surface modification with meso-2,3-dimercaptosuccinic acid [26]. After that, melamine and glutaraldehyde were employed to generate dendrimer-like polymers and were grafted onto carboxylated magnetic nanoparticles, resulting in the formation of the new support MG-DMNPs. Then, the resulting particles were used to immobilize processive endo-glucanase EG5C-1. The immobilization conditions were optimized, and the physicochemical properties of immobilized EG5C-1 were studied. Finally, the hydrolysis efficiency and reusability of the MG-DMNP/EG5C-1 biocomposites were determined using CMC and filter paper as substrates.

## 2. Materials and Methods

### 2.1. Materials

Carboxymethylcellulose sodium (CMC) and 3,5-dinitrosalicylic acid (DNS) were obtained from Alfa Aesar (Tianjin, China). Melamine, glutaraldehyde, and meso-2,3-dimercaptosuccinic acid (DMSA) were provided by Aladdin Reagent (Shanghai, China). Whatman filter papers were obtained from GE Healthcare (Shanghai, China). Standard cello-oligosaccharides were ordered from Megazyme (Wicklow, Ireland). The BCA protein assay kit was acquired from Sangon Biotech (Shanghai, China). All remaining chemicals used were of analytical grade.

### 2.2. Preparation and Surface Modification of Magnetic Nanoparticles

Fe_3_O_4_ nanoparticles were prepared by a chemical co-precipitation method reported earlier with slight modification [27]. Bivalent ferric chloride and trivalent ferric chloride were dissolved in distilled water at a molar ratio of 1:2 under N_2_ protecting, followed by the slow addition of enough ammonia aqueous solution with vigorous stirring. The resulting black precipitate was washed several times with deionized water. The final magnetite nanoparticles were dispersed in deionized water with pH 3.0 and oxidized into more stable MNPs (γ-Fe_3_O_4_) by air at 90 °C. Then, these nanoparticles were dispersed in deionized water, and the pH was adjusted to 2.7. Subsequently, DMSA was dissolved in dimethyl sulfoxide (DMSO) and added in MNP solution with continuous stirring. After 5 h at room temperature for the reaction, the products were collected with a magnet and dispersed in (CH_3_)_4_NOH solution, then the mixture was adjusted to a pH of 10. The DMSA-coated MNPs (DMNPs) were obtained after the pH of the solution was adjusted to neutral. Then, the nanoparticles were washed with distilled water by magnetic separation to remove chemical residuals. Subsequently, melamine–glutaraldehyde dendrimers were grafted onto the surface of DMNPs according to the previous method with several modification. The solution of melamine (100 mL, 3 mg/mL) was mixed with 120 mL solution of 5% glutaraldehyde. Then, 100 mg of DMNPs were redissolved in the solution above and kept at 40 °C with a shaking speed of 200 rpm for 12 h. The modified particles (MG1-DMNPs) were washed with ethanol three times and stored at room temperature in a 2% glutaraldehyde solution for further use. The synthesis of MG2-DMNPs to MG4-DMNPs was as follows: 100 mg MG1-MNPs were dispersed in 100 mL of a melamine dimethyl sulfoxide solution (6 mg/mL) at 40 °C with a shaking speed of 200 rpm for 12 h. The excess dimethyl sulfoxide was used to wash the carrier to remove unconnected melamine with glutaraldehyde on the carrier surface. Then, 100 mg of MG1-DMNPs were mixed with 120 mL of a 10% glutaraldehyde solution and kept under the same conditions for 12 h. After that, it was mixed with 100 mL of a 2% glutaraldehyde solution to coat the aldehyde group. This carrier was named MG-CA-MNPs2. Similarly, 100 mg of MG2-DMNPs were dispersed in 100 mL of a 12 mg/mL melamine dimethyl sulfoxide solution at 40 °C for 12 h. Then, the carrier was washed with dimethyl sulfoxide and mixed with 120 mL of a 20% glutaraldehyde solution at 40 °C for 12 h. MG3-DMNPs were obtained. At 40 °C, 150 mg of MG3-DMNPs were evenly dispersed in 200 mL of a 12 mg/mL melamine dimethyl sulfoxide solution for 12 h. After washing the carrier with dimethyl sulfoxide, it was mixed with 120 mL of a 40% glutaraldehyde solution at 40 °C for 12 h. Then, GM4-DMNPs were obtained. The carrier was subsequently washed with dimethyl sulfoxide solution and hot water and stored in a 2% glutaraldehyde solution. 

### 2.3. Immobilization of EG5C-1 on Carrier MG-DMNPs

The construction, expression, and purification of C-terminally His-tagged EG5C-1 were carried out according to our previous methods [28]. The purified enzyme was assayed by SDS-PAGE and freeze-dried for storage. The carriers of MG1-DMNPs to MG4-DMNPs (100 mg) were dispersed in 100 mL of phosphate buffer (pH 6.0). Then, a certain amount of EG5C-1 solution was added into the suspension and stirred at room temperature. The EG5C-1-bonded MG-DMNPs were magnetically separated and washed several times with the same buffer, and then lyophilized for later use. All the supernatants were collected for calculating the residual protein content. The immobilized conditions, including the concentrations of EG5C-1 (2–10 mg/mL), pH values (5–8), immobilization time (1–6 h), and the number of tris (2,4,6-trimethoxyphenyl) phosphonium bromide modifications (1–4), were optimized one by one. The enzyme adsorption amount and activity recovery rate were calculated using Equation (1),

(1) $$Load\;capacity=\frac{W1-W0}{M};\;Activity\;recovery=\frac{A1}{A2}\times 100\%$$

where W1 is the protein content of the free enzyme under the same conditions, W0 is the protein content in the supernatant after immobilization, M is the mass of the carrier material, and A1 and A2 are the activities of the immobilized and free EG5C-1, respectively.

### 2.4. Protein Concentration Measurements and Enzyme Activity Assay

The BCA method was used to determine the protein concentration of the original enzyme solution before immobilization, the supernatant after immobilization, and washing solution [29]. The enzyme activities of immobilized and free EG5C-1 were determined using the carboxymethyl cellulose (CMC) method and the amounts of reducing sugar released were analyzed using the 2,4-dinitrosalicylic acid (DNS) method [30]. Specifically, the substrate was preheated at 50 °C in a water bath for 10 min, and free and immobilized enzymes were added separately into the substrate solution. To maintain the same enzyme content, equal amounts of free and immobilized enzymes were added based on the previously measured immobilized protein content [31]. After a 10 min reaction, DNS was added, the immobilized enzyme was magnetically separated, and the supernatant was measured using a spectrophotometer at 562 nm to determine enzyme activity units, defined as the amount of enzyme required to release 1 μmol of reducing sugar per minute [32]. 

### 2.5. Characterization

The surface morphologies of Fe_3_O_4_ and MG3-DMNPs, as well as EG5C-1/MG3-DMNP biocomposite, were observed by field emission scanning electron microscopy (FEI, Hillsboro, OR, USA). Fourier transform infrared (FTIR) spectroscopy was performed using a 100 spectrometer (Perkin Elmer, Shanghai, China) to scan in the wavelength range of 500–4000 cm^−1^. Thermal gravimetric analysis (Netzsch, Shenzhen, China) was conducted in a nitrogen atmosphere at a heating rate of 10 °C/min from 20 to 600 °C. X-ray photoelectron spectroscopy (XPS) analysis was performed on a 250xi instrument (Thermo Scientific, Shanghai, China) for element and energy analysis.

### 2.6. Biochemical Properties

The effects of temperature on the activities of free and immobilized EG5C-1 were determined in a phosphate buffer solution with a pH of 6 at temperatures ranging from 30–80 °C [33]. The effects of pH on the activities of free and immobilized cellulase were detected at 50 °C using buffer solutions with pH values ranging from 5–8. The pH stabilities of free and immobilized EG5C-1 were evaluated by measuring the residual activity after incubating in different buffer solutions, including citric acid/sodium citrate buffer (pH 5–6) and 50 mmol/L sodium phosphate buffer (pH 6–8) for 3 h. To determine the thermal stability, the thermal inactivations of free and immobilized EG5C-1 were measured by incubating at 45 °C for 8 h. After incubation, the residual activities of free and immobilized enzyme were measured, and relative activities were calculated with Equation (2).

(2) $$\mathrm{Relative}\;\mathrm{activities}=\frac{A1}{A2}\times 100\%$$

where A1 and A2 are the activities of the residual activities and original activities, respectively.

### 2.7. Saccharification of Filter Paper by the Immobilized EG5C-1

To determine the catalytic efficiency of immobilized EG5C-1, filter paper was used as substrate of saccharification. As mentioned earlier, the substrate concentration was 2% (*w*/*v*) and the enzyme loading was 10 FPU/g. Enzymatic hydrolysis was performed in phosphate buffer (pH 6) at 50 °C and 150 rpm for 36 h with free EG5C-1 as a control [34]. Samples were extracted at different catalytic time points, and the hydrolysis of the substrate was recorded. The released reducing sugar was measured using the DNS method [32].

### 2.8. Reusability Assay of the Immobilized EG5C-1

To test the reusability of immobilized enzymes, EG5C-1/MG3-DMNP biocomposite was incubated with substrate in phosphate buffer (pH 6) and shaken continuously at 50 °C for 12 h. At the end of the enzymatic hydrolysis process, the biocomposite was separated by a magnet and washed by buffer (pH 6). For the following cycle, the recovered immobilized EG5C-1 was re-suspended in fresh substrate solution for a new hydrolysis [35]. The content of reduced sugar in the supernatant was measured for each round to determine the repeatability of immobilized EG5C-1.

## 3. Results and Discussion

### 3.1. Immobilization of EG5C-1 onto Melamine–Glutaraldehyde Magnetic Nanoparticles

**Scheme 1:**
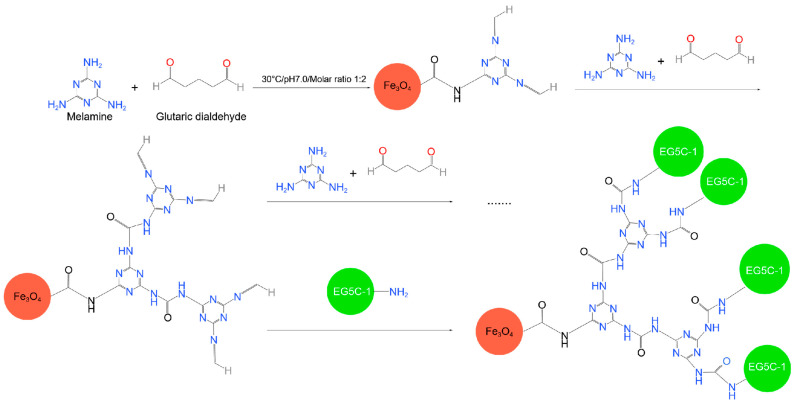
Schematic diagram of carrier material modification and enzyme immobilization.

**Figure 1:**
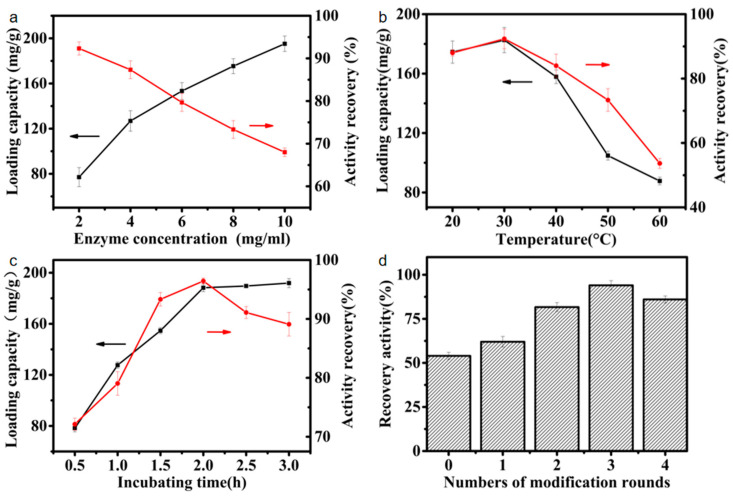
Effects of initial enzyme concentration (**a**), temperature (**b**), and incubation time (**c**) on loading capacity and activity recovery of the MG-DMNP/EG5C-1 and effects of number of modification rounds on activity (**d**).

Firstly, MNPs were synthesized by coprecipitation and melamine–glutaraldehyde dendrimer-like polymer was grafted onto the surface of the carriers one after another. This synthesis process was schematically illustrated in Scheme 1. The purified EG5C-1 was used for immobilization. The free EG5C-1 had an enzyme activity of approximately 131 U/mg on CMC. In this study, we tested the direct immobilization of enzymes on the DMNPs and the immobilization of enzymes on the DMNPs with different modification rounds of melamine–glutaraldehyde. We found that DMNP materials modified with melamine–glutaraldehyde showed significant improvement in protein loading and activity recovery. During the enzyme immobilization process, reaction conditions have a significant impact on the catalytic ability and activity recovery of the enzyme. Therefore, the effect of EG5C-1 dosage, solution pH, and catalytic time on the immobilization of EG5C-1 on CMC was studied to achieve the optimal immobilization conditions. As shown in Figure 1a, the immobilization amount and activity recovery of enzymes at different concentrations were studied. When the EG5C-1 concentration for immobilization increased from 2.0 mg/mL to 10 mg/mL, the loading amount increased from 77.0 to 195.2 mg/g, while the activity recovery rate decreased from 92.3% to 68%. One possible explanation for this phenomenon is that excessive enzyme loading may produce severe spatial hindrance, thereby limiting the entry of EG5C-1 catalytic sites. The immobilization of 10 mg/mL EG5C-1 resulted in a specific activity of only 89.1 U/mg, which was significantly lower than the activity of 121.0 U/mg at 2 mg/mL, consistent with this explanation. Similar conclusions have been reported for other immobilized enzyme systems [36,37,38,39]. Considering the loading capacity and activity recovery, the optimal immobilized enzyme concentration was approximately 6.0 mg/mL.

Figure 1b shows the protein loading and activity recovery of the immobilized enzyme at different solution temperatures ranging from 30 °C to 70 °C, to investigate the optimal reaction temperature for EG5C-1 immobilized on MNPs. The activity recovery of EG5C-1-MNPs kept increasing within the temperature range of 30 °C to 60 °C, reaching a maximum at 60 °C with a protein loading of 188 mg/g. When the temperature reached 70 °C, both activity recovery and loading capacity slightly decreased to 87% and 174.7 mg/g, respectively. Therefore, the optimal temperature for EG5C-1 immobilization was 60 °C. Figure 1c shows the relationship between protein loading, activity recovery, and immobilization time. Under the conditions of 40 °C and pH 7.0, both protein loading and specific activity increased with increasing immobilization time, reaching equilibrium at a loading capacity of 188 mg/g and activity recovery of 96% within 2 h. Further incubation time (up to 3 h) did not improve the loading capacity of EG5C-1. However, although the activity recovery peaked after 2 h of immobilization, the activity gradually decreased with further immobilization time, possibly due to the deactivation of EG5C-1 over time.

We also tested the effect of the number of melamine–glutaraldehyde (MG) modifications on activity recovery (Figure 1d). When EG5C-1 was adsorbed onto regular MNPs modified only with meso-2,3-dimercaptosuccinic acid, the activity recovery was only about 80%, which is within the normal range compared to previous studies [28]. Regarding the number of modifications, the experiment showed that the material with three modifications had the highest activity and adsorption capacity, with an adsorption capacity of 180 mg/g and an activity recovery of 94%. Although, theoretically, excessive modifications can increase the binding sites, in reality, the material itself will undergo self-crosslinking during the modification and immobilization processes [40]. The first scenario is the crosslinking of the material itself. When melamine is connected, the amino group on one end is crosslinked with another successfully modified aldehyde group. The second scenario is the fixation of enzyme sites with multiple carrier particles during the immobilization of enzymes (Figure S1). This results in the crosslinking of enzymes with multiple carrier materials, which affects the enzyme catalytic activity due to possible alterations in the spatial conformation of the enzymes. During the experiment, we observed that the immobilized enzyme material starting from the fourth modification round would exhibit obvious interconnection in morphology (Figure S2). The carrier iron oxide particles changed from a granular shape to a colloidal complex form, and the carrier could not form a dispersion state, even after several washes. This is also reflected in the SEM images. By comparing the scanning electron microscope images of Fe_3_O_4_ and MG4-DMNPs with the same size, it can be seen that the MG4-DMNPs are obviously aggregated into blocks (Figure S3). A similar phenomenon was also observed by Wang et al. [41]. This self-crosslinking might affect the adsorption of enzyme proteins and the enzyme catalytic activity. For example, in our data, the increase in protein loading capacity of the carrier with three modifications was not as significant as that between the first and second modifications, indicating the influence of self-crosslinking. However, the modified material still maintained its superparamagnetic characteristics, and the advantages of iron oxide in immobilizing enzymes using an external magnet were not affected [42]. With four or more modifications, the adhesion became more severe, and the magnetic properties were significantly reduced. In the presence of an external magnet, the adsorption rate was significantly lower than that of unmodified samples or those after one or two rounds of modification, which can also be reflected in the VSM characterization. The increase in protein loading capacity was very limited, and in some cases, due to severe self-adhesion, the results were worse than those of three rounds of modification (Figure 1d). However, the activity recovery of immobilized enzymes did not change significantly with four or more modifications, which also conforms to the characteristics of enzyme mimics. The catalytic effect of immobilized enzymes is essentially due to the enzyme, and the modification method and carrier peroxidase properties only play an auxiliary role. Therefore, we conclude that three rounds of modification are the upper limit of this modification method, achieving high enzyme activity and loading capacity. Excessive modification will result in self-crosslinking, which affects the catalytic efficiency [7,43]. Our study showed that iron oxide nanoparticles, as a carrier, coupled with melamine–glutaraldehyde dendrimer-like modification, significantly increased the covalently bound protein amount and effectively improved the activity recovery of the immobilized enzyme. Under the optimal conditions of 60 °C, pH 7.0, and a reaction time of 5 min, the specific activity of the optimal immobilized enzyme reached 92% of that of the free enzyme. The possible reasons for this are the peroxide properties of iron oxide nanoparticles and the catalytic effect of Schiff base. Compared with the traditional amide bond, the Schiff base bond has additional electrons, which is conducive to ion exchange during the reaction. However, the precise mechanisms of action of the Schiff bases are not yet fully understood and, therefore, continue to deserve further investigations [44]. In previous studies, Schiff base complexes have been used as catalysts for their high biological activity [45,46]; we also found that the use of Schiff base increased the enzyme activity [47].

### 3.2. Characterization

**Figure 2:**
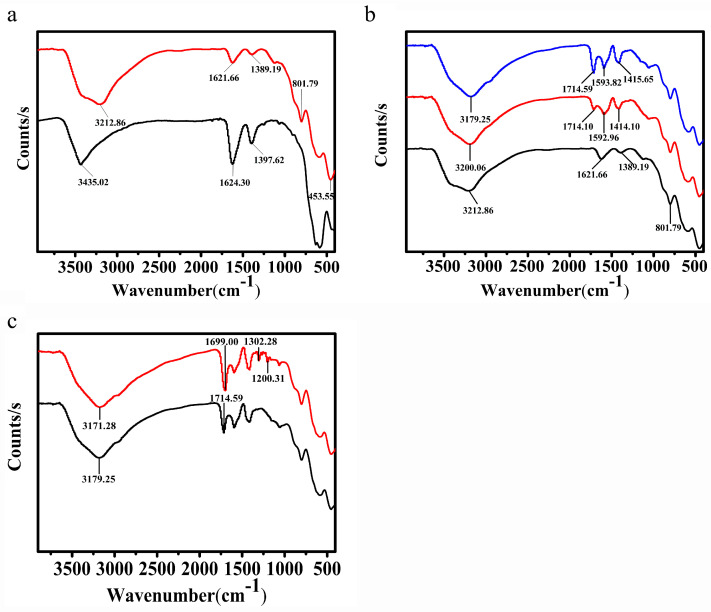
FTIR spectra of unmodified Fe_3_O_4_ (black) and DMNPs (red) (**a**). DMNPs (black), MG1-DMNPs (red), and MG3-DMNPs (blue) (**b**). MG3-DMNPs (black) and MG-DMNP/EG5C-1 (red) (**c**).

The FTIR spectra of various materials were studied, as shown in Figure 2a. The peak at 3435 cm^−1^ in the unmodified spectrum shifted to around 3200 cm^−1^ after surface modification with meso-2,3-dimercaptosuccinic acid, and a new peak appeared at 801 cm^−1^, which was attributed to the surface -OH and carboxyl-CO vibrations formed by meso-2,3-dimercaptosuccinic acid modification. The clear trend in the changes shown in the figure indicates the successful modification with meso-2,3-dimercaptosuccinic acid. Other important absorption bands appeared in the spectra of the second group (Figure 2b) at 1714 cm^−1^ and 1415 cm^−1^, which were attributed to the Schiff base N=C bending vibration and N−C stretching vibration produced by 1–3 rounds of modification with melamine–formaldehyde [47]. The peak at 1592 cm^−1^, which was present in all spectra, was attributed to the typical stretching vibration of C=O. In Figure 2c, two peaks appeared at 1302.3 and 1200.3 cm^−1^ in the FTIR spectrum after EG5C-1 was attached to the polymer, which were related to the C=O and N−H vibrations in the protein, indicating that EG5C-1 was successfully immobilized on the MG3-DMNPs [48].

**Figure 3:**
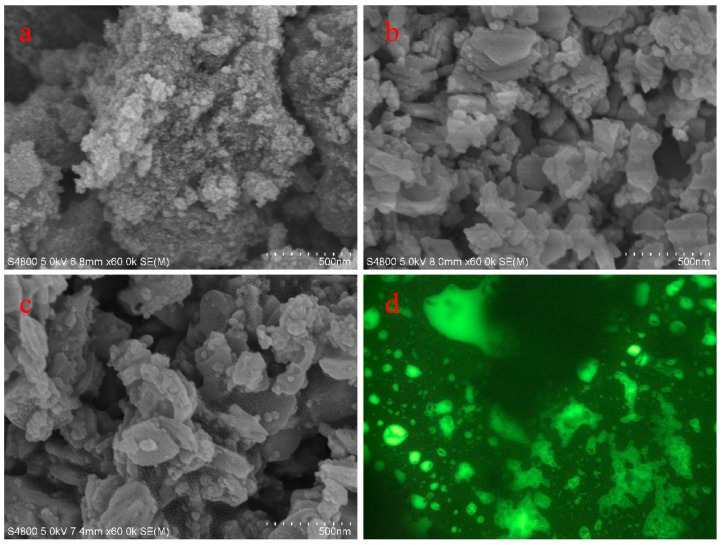
SEM images of Fe_3_O_4_ (**a**), MG3-DMNPs (**b**), and MG-DMNP/EG5C-1 (**c**). Fluorescent protein image of MG-DMNP/EG5C-1 (**d**).

After collecting scanning electron microscopy (SEM) and fluorescent protein image data (Figure 3), the modification methods for functionalizing MNPs were determined by detecting changes in overall morphology [49]. It was found that there were slight changes in morphology observed after modification with meso-2,3-dimercaptosuccinic acid and melamine–glutaraldehyde (Figure 3a,b). The original state of MNPs was granular and dispersed. However, the modified particles formed smooth polymers on the surface, including flat surface wrapping and circular complexes. After immobilizing the enzyme, the overall morphology sharpness decreased again, and obvious enzyme attachment could be observed (Figure 3c). Fluorescent proteins were immobilized on the material and the surface morphology was observed using an upright microscope and a 488 nm laser (Figure 3d), which proved that the protein was successfully immobilized.

**Figure 4:**
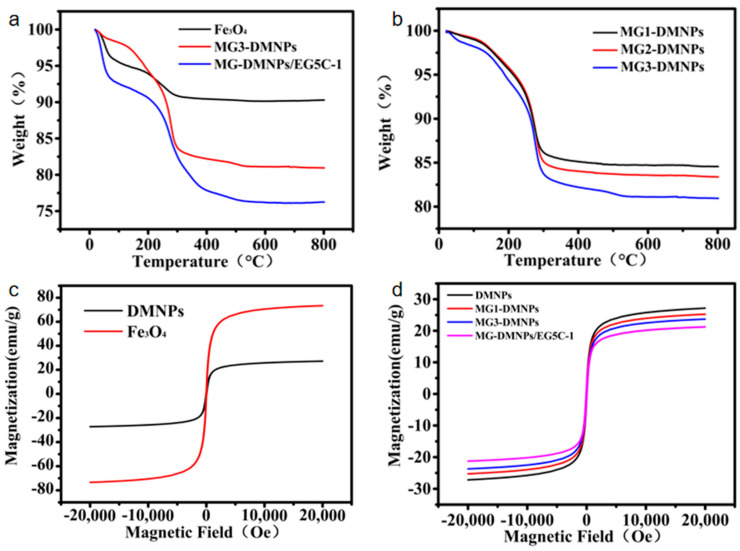
TGA curves of Fe_3_O_4_, MG3-DMNPs, and MG-DMNP/EG5C-1 (**a**), and MG1-DMNPs, MG2-DMNPs, and MG3-DMNPs (**b**). VSM magnetic hysteresis loops of Fe_3_O_4_ and DMNPs (**c**), DMNPs, MG1-DMNPs, and MG3-DMNPs, as well as MG-DMNP/EG5C-1 (**d**).

Using TGA technology, the thermal decomposition process of Fe_3_O_4_, MG3-DMNPs, and MG-DMNP/EG5C-1 biocomposite materials was studied (Figure 4a). The TGA curves showed two main stages of decomposition. The Fe_3_O_4_ carrier and immobilized enzyme carrier degraded by about 5% and 10%, respectively, in the range of 0–200 °C, which was estimated to be due to the dehydration of surface-bound water molecules and loosely bound enzymes [10,50]. In the range of 200–300 °C, compared with Fe_3_O_4_, MG3-DMNP material and MG-DMNP/EG5C-1 biocomposite material both showed some degradation, and the derivative of the degradation curve increased significantly, indicating the successful modification of melamine–glutaraldehyde crosslinking. The residual weight of the materials decreased in the order of different modification rounds (Figure 4b), verifying the success of multi-round modification. After 300 °C, the TGA curve of the MG-DMNP/EG5C-1 biocomposite material indicated that the enzyme Schiff base crosslinking product was completely burned in the air flow. Compared with MG-DMNP/EG5C-1, the weight loss of MG3-DMNPs was less, confirming the existence of protein in MG-DMNP/EG5C-1 biocomposites and indicating the successful immobilization of the enzyme [7].

The magnetic properties of the nanocomposites were studied using the VSM method. Figure 4c shows the VSM magnetic hysteresis loops of Fe_3_O_4_ and the DMNPs. The magnetic hysteresis measurement of the carrier material was performed at room temperature with an external magnetic field of ~20,000 kV. As shown in the figure, the M (H) hysteresis loop is completely reversible, indicating that all MNPs have superparamagnetic properties. Both types of particles exhibit relatively high magnetization, and their magnetization strength is sufficient for magnetic separation induction using conventional magnets. The reversibility of the hysteresis loop confirms that the nanoparticles do not aggregate in the magnetic field. Moreover, the decrease in magnetization strength after meso-2,3-dimercaptosuccinic acid modification, increasing modification rounds, and enzyme immobilization also confirms the successful immobilization of the materials [33,43,51].

**Figure 5:**
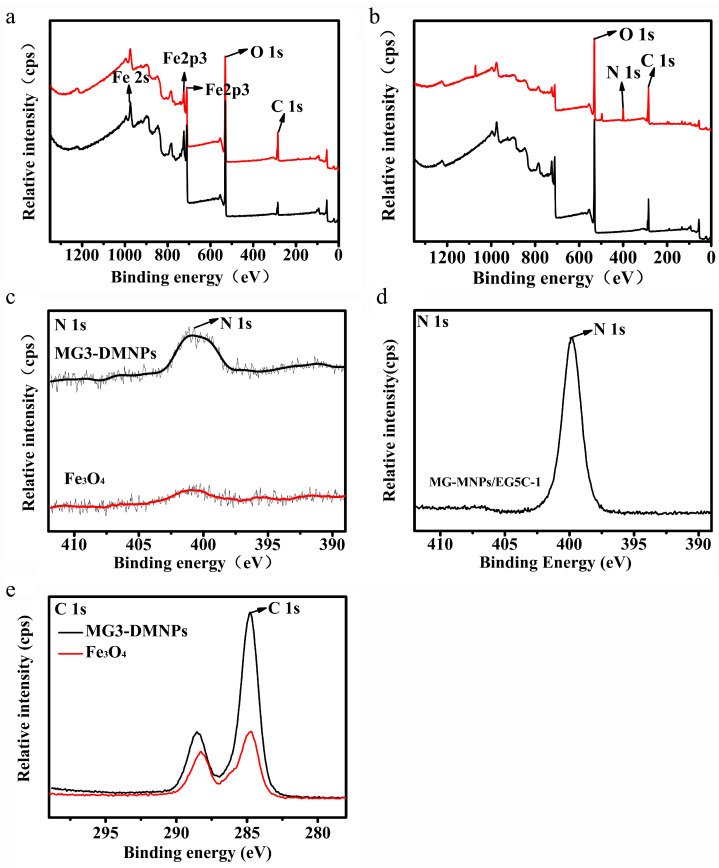
XPS spectra of Fe_3_O_4_ (black) and DMNPs (red) (**a**), and MG3-DMNPs (black) and MG-DMNP/EG5C-1 (red) materials (**b**). N 1s spectrum of MG3-DMNPs and Fe_3_O_4_ materials (**c**), N 1s spectrum of MG-DMNP/EG5C-1 (**d**), and C 1s spectrum of MG3-DMNPs and Fe_3_O_4_ materials (**e**).

As shown in Figure 5a, it can be seen that Fe_3_O_4_ material has elements such as Fe 3s, Fe 3p, Fe 2p1, Fe 2p3, and a very high O1s peak. This is a natural result of the properties of the iron oxide peroxide material itself. During the process, the peak of the N element has been very low [28,42]. After three rounds of modification, an N 1s peak appeared at 400 eV (Figure 5c), which was due to Schiff base modification until EG5C-1 was fixed, resulting in a more pronounced N peak (Figure 5b,d), proving the successful fixation of the enzyme. In addition, the decrease in Fe 3p and Fe 2p peaks with the increase in modification times also confirms the success of the modification [52]. With meso-2,3-dimercaptosuccinic acid and complex modification, the peak of the C element significantly increased and appeared at 284.8eV and 288.7eV, which is the effect of the regular O-C=O group and C-H group (Figure 5e).

### 3.3. Biochemical Properties of Immobilized EG5C-1

**Figure 6:**
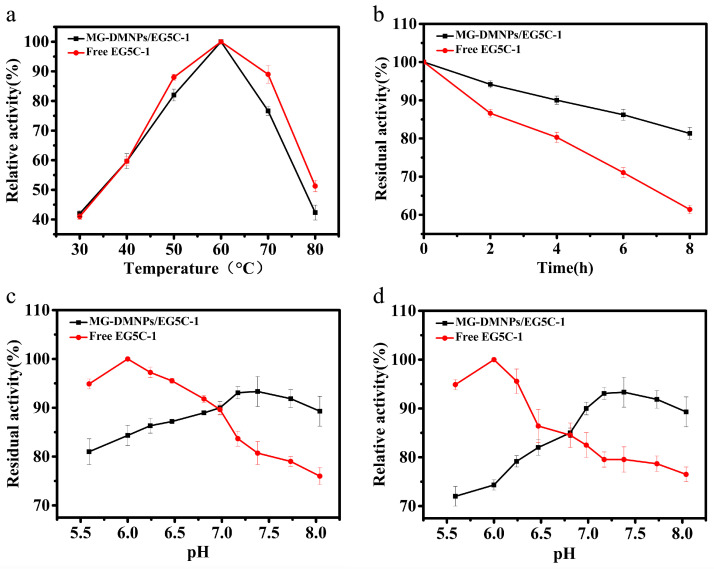
Effect of temperature on the activity of free EG5C-1 and MG-DMNP/EG5C-1 (**a**). Thermal inactivation of free EG5C-1 and MG-DMNP/EG5C-1 (**b**). Effect of pH on the activity of free and immobilized EG5C-1 (**c**). pH stability of free and immobilized EG5C-1 (**d**).

The temperature and pH are the main factors that affect the catalytic activity of enzymes. Therefore, we studied the effects of temperature and pH on catalytic activity and stability of free and immobilized enzymes. As shown in Figure 6a, both free and immobilized EG5C-1 showed the highest catalytic activity at 60 °C, indicating that immobilization did not alter the optimal temperature of enzyme EG5C-1.

The thermal deactivation of free and immobilized enzyme samples was tested at 50 °C (Figure 6b). The activity of free EG5C-1 decreased much faster than that of the immobilized enzyme with extension of the incubation time. Free EG5C-1 remained at only 61.2% of the activity after 8 h incubation, while immobilized EG5C-1 demonstrated 83.4% of its original activity at the same conditions. The optimal pH values for both free and immobilized EG5C-1 were studied within the pH range of 5.5 to 8.0 (Figure 6c). The free enzyme maintained over 90% activity at pH 5.6–6.5, with the highest initial activity recorded at pH 6.0, and a decrease in activity was observed within the pH range of 6–8. The trend for immobilized enzyme was opposite, with only about 81% enzyme activity recovery at pH 5.5, but with an increase in activity as pH increased. At the highest pH value of 6.0 for free enzyme activity, immobilized enzyme activity recovery reached 84%, and the highest recovery rate was 93% at around pH 7.0, which is different from conventional immobilization materials [53]. As speculated, the main reason for this phenomenon is that Schiff base is more easily decomposed into the aldehyde and amine formed during synthesis than the amide bond in acidic environments, as mentioned in previous studies [43,47]. The excess aldehyde and amine substances produced can affect enzyme activity and even hinder the binding of enzyme and carrier. In terms of pH stability, immobilized enzymes lost a large amount of activity below pH 7.0, but showed good performance compared to free enzymes above pH 7.0 (Figure 6d).

Using CMC and filter paper as substrates, the kinetic parameters of immobilized EG5C-1 and free EG5C-1 were determined, and the specific data are shown in Table 1. The apparent K_m_ values of immobilized EG5C-1 for CMC and filter paper substrates were 8.2 and 86.4 mg/mL, respectively, which were higher than the data of the free enzyme, indicating that the affinity of immobilized EG5C-1 for substrates was slightly lower than that of the free enzyme [31,38]. The contact area between the immobilized enzyme and the substrate was reduced after immobilization, which is consistent with the trend presented in many previous studies [24]. The turnover numbers (k_cat_) of immobilized EG5C-1 for CMC and filter paper hydrolysis were 232.8 s^−1^ and 2.8 s^−1^, respectively, which were higher than the measured values for the free enzyme (196.2 s^−1^ and 2.7 s^−1^). However, due to a lower affinity of the immobilized enzyme towards two substrates in comparison to the free one, the catalytic efficiency (k_cat_/K_m_) of the immobilized enzyme towards CMC and filter paper was lower than that of the free one. In general, the catalytic efficiency will significantly decrease when the immobilized cellulase hydrolyzes insoluble or solid-form substrates, due to the diffusion limitation of spatial sites, substrates, and products, and the loss of enzyme flexibility [23,54]. However, the catalytic efficiency (k_cat_/K_m_) of the immobilized enzyme in this work did not decrease significantly, especially when the substrate is insoluble or in solid form.

### 3.4. Hydrolysis of Filter Paper Using Free and Immobilized EG5C-1

**Figure 7:**
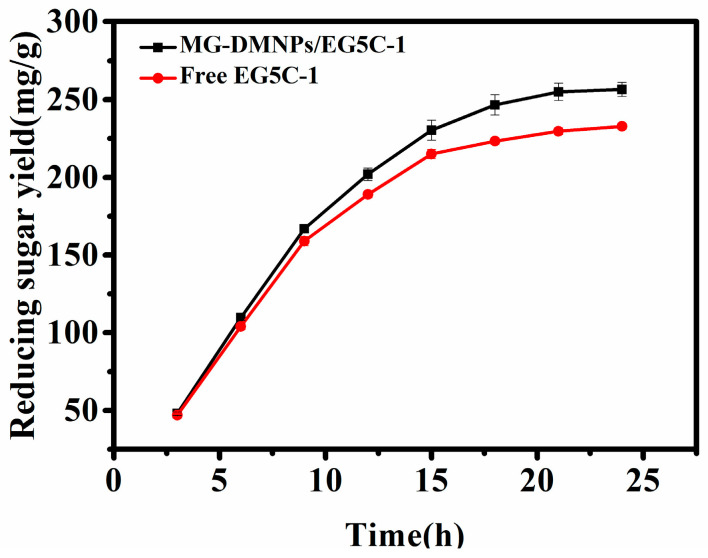
Reducing sugar yield of filter paper by free EG5C-1 and MG-DMNP/EG5C-1.

In this study, laboratory quantitative filter paper was used as a substrate to investigate the saccharification of free and immobilized EG5C-1. Under the conditions of pH 6.5 and 50 °C, 100 mg of immobilized enzyme with an enzyme loading of 180 mg/g and an equal concentration of 6 mg/mL of free enzyme (3 mL) were added to a hydrolysis reaction for 24 h. The maximum reducing sugar yield of free EG5C-1 was 256.6mg/g filter paper, while the reducing sugar yield of immobilized EG5C-1 was 232.4 mg/g (Figure 7). The immobilized enzyme maintained 90.6% hydrolysis efficiency. The filter paper hydrolysis yield of immobilized EG5C-1 was slightly lower than that of CMC, which may be related to unavoidable mass transfer limitations or poor enzyme-substrate contact opportunities.

### 3.5. Reusability of Immobilized EG5C-1

**Figure 8:**
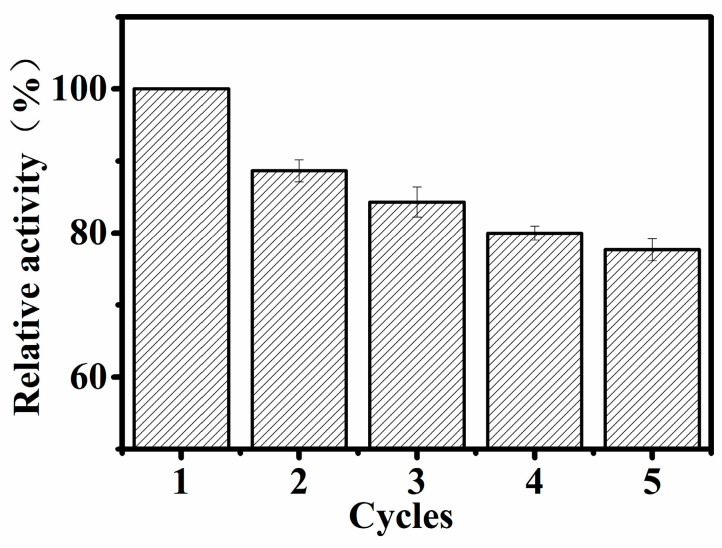
Reusability of immobilized EG5C-1 using CMC as the substrate.

Figure 8 shows the reusability of immobilized EG5C-1. The operational stability of immobilized enzymes is a key factor in reducing costs in practical applications, and the number of times they can be reused is an important criterion [4,16,23,47]. Using CMC as the substrate, the usability of immobilized EG5C-1 was studied by repeating the catalytic cycle. The significant advantage of magnetic nanoparticles can be utilized by separating the immobilized carrier and product using an external magnet after each catalysis, and then measuring the reducing sugar content of the product and simply washing away any residual product on the surface before using the carrier in the next round of reaction [6,10,19]. This process can accurately test the specific reducing sugar yield of each round of reaction. As shown in the figure, after the first reaction, immobilized EG5C-1 lost 13% of its CMC hydrolysis ability. However, after five cycles, the CMC hydrolysis ability of immobilized EG5C-1 remained at nearly 80%. The trend in the figure shows that the loss of catalytic ability in the last four cycles was less than that in the first cycle. The significant decrease in catalytic ability in the first cycle was mainly due to some EG5C-1 molecules being weakly bound by physical adsorption, which are easy to detach. These weakly bound molecules are easily detached after the first round of reaction, and the reaction protein decreases in activity in a certain proportion in the later cycles, which is a deactivation phenomenon that conforms to the trend in the figure [28]. Overall, our results demonstrate that physical adsorption is a relatively weak bond compared to the Schiff base covalent bond between EG5C-1 and melamine–formaldehyde magnetic nanoparticles, which is not sufficient to enable EG5C-1 to be used on magnetic nanoparticles for a long time. Similar observations have been reported in other studies using cellulase [55], chitosan polymer immobilized chitosanase [56], and laccase [10], where the retention rate was only about 50–60% after 3–5 cycles.

## 4. Conclusions

In this study, magnetic nanoparticles with dendrimer-like polymers of melamine–formaldehyde modified with meso-2,3-dimercaptosuccinic acid were used as a carrier to immobilize endoglucanase EG5C-1. Meso-2,3-dimercaptosuccinic acid was first fixed to increase the surface carboxyl groups, which facilitated the complexation with Schiff base. After several rounds of modification, the enzyme was immobilized at room temperature, with an optimal loading capacity of approximately 195 mg/g where more than 90% of the activity was recovered. Compared with the free enzyme, immobilized EG5C-1 exhibited improved thermal stability and increased tolerability over a broad pH range. After five cycles of use, the hydrolysis rate remained close to 80% of the initial value. This carrier and modification method successfully improved the activity recovery of cellulase catalysis beyond that of the free enzyme itself. The system developed in this study provides an attractive alternative for enzymatic hydrolysis of cellulose biomass.

## Figures and Tables

**Table 1:** Apparent kinetic parameters of free and immobilized EG5C-1.

Substrate	Enzyme	K_m_ (mg/mL)	k_cat_ (s^−1^)	k_cat_/K_m_ (mL/mg/s)
CMC	Free EG5C-1	5.3	196.2	37.0
	Immobilized EG5C-1	8.2	232.8	28.4
Filter paper	Free EG5C-1	77.5	2.7	0.035
	Immobilized EG5C-1	86.4	2.8	0.033

## Data Availability

All relevant data are within the manuscript and Supplementary Materials.

## Supplementary Materials

The supporting information can be downloaded at: https://www.mdpi.com/article/10.3390/nano14040340/s1.
