# Supplementary material for: Immobilization and Characterization of a Processive Endoglucanase EG5C-1 from Bacillus subtilis on Melamine–Glutaraldehyde Dendrimer-Functionalized Magnetic Nanoparticles
Source: Nanomaterials (Basel). 2024 Feb 9;14(4):340. doi: 10.3390/nano14040340 (PMC10891739; doi:10.3390/nano14040340)
Supplement: Supplementary file 1 [file nanomaterials-14-00340-s001.zip › nanomaterials-2833446-supplementary.pdf]

Supplementary Materials

Immobilization and Characterization of a Processive Endoglucanase EG5C-1 from *Bacillus subtilis* on Melamine–Glutaraldehyde Dendrimer-Functionalized Magnetic Nanoparticles

Xiaozhou Li [1], Jie Chen [1], Bin Wu [1], Zhen Gao [1,*] and Bingfang He [2]

[1] College of Biotechnology and Pharmaceutical Engineering, Nanjing Tech University, Nanjing 211800, China; 202161218101@njtech.edu.cn (X.L.); 202261118054@njtech.edu.cn (J.C.); wubin1977@njtech.edu.cn (B.W.)
[2] School of Pharmaceutical Sciences, Nanjing Tech University, Nanjing 211800, China; bingfanghe@njtech.edu.cn
* Correspondence: gaozhen@njtech.edu.cn

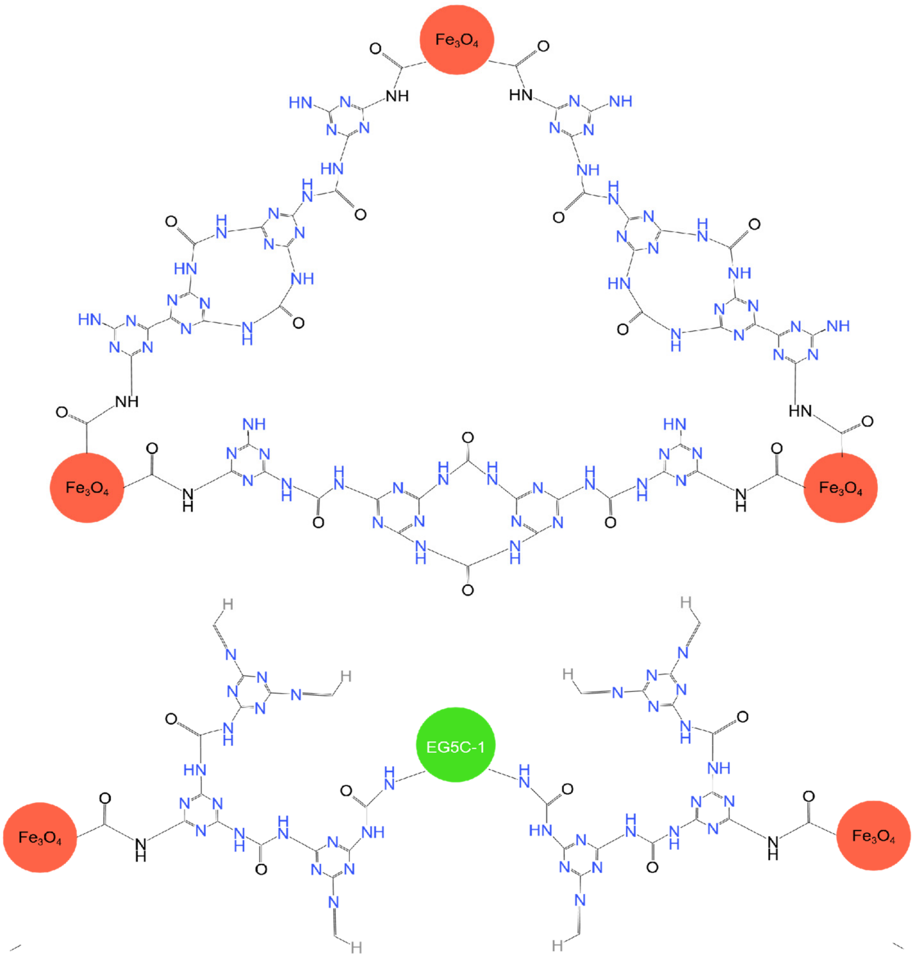

Figure S1. Chemical structure diagram of adhesion of melamine and glutaraldehyde between MG3-DMNPs and MG4-DMNPs and fixation of enzyme sites with multiple carrier particles.

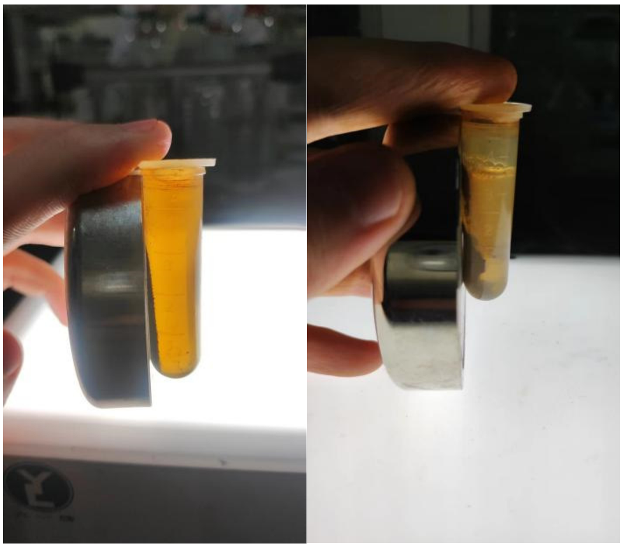

Figure S2. External magnetic attraction diagram of DMNPs and MG4-DMNPs.

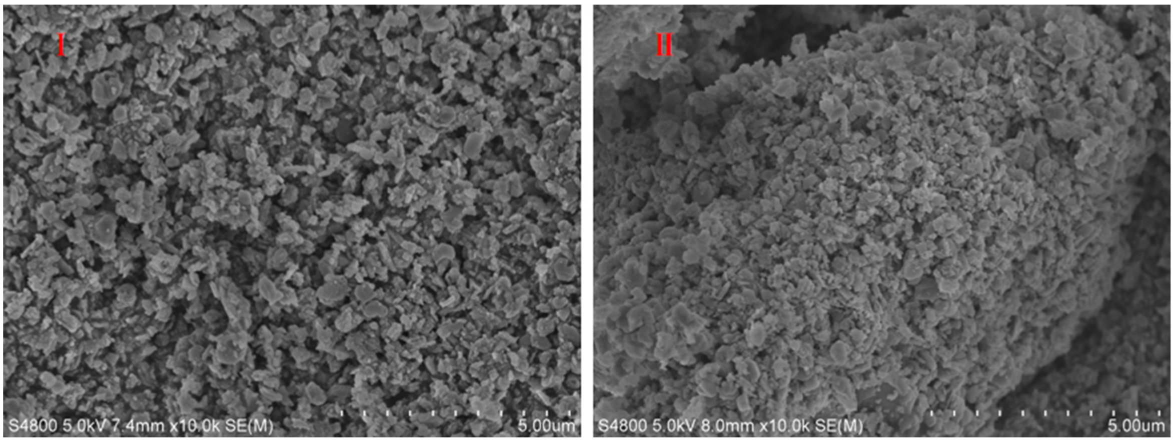

Figure S3. SEM image of Fe_3O_4(I), and MG4-DMNPs(II).
